# Customized 3D-Printed Mesh, Membrane, Bone Substitute, and Dental Implant Applied to Guided Bone Regeneration in Oral Implantology: A Narrative Review

**DOI:** 10.3390/dj12100303

**Published:** 2024-09-25

**Authors:** Federica Di Spirito, Francesco Giordano, Maria Pia Di Palo, Cosimo Ferraro, Luigi Cecere, Eugenio Frucci, Mario Caggiano, Roberto Lo Giudice

**Affiliations:** 1 Department of Medicine, Surgery and Dentistry, University of Salerno, Via S. Allende, 84081 Baronissi, SA, Italy; frgiordano@unisa.it (F.G.); mariapia140497@gmail.com (M.P.D.P.); c.ferraro17@studenti.unisa.it (C.F.); luigi.cecere97@gmail.com (L.C.); eugenio.frucci@gmail.com (E.F.); macaggiano@unisa.it (M.C.); 2 Department of Human Pathology in Adulthood and Childhood “G. Barresi”, University Hospital “G. Martino” of Messina, Via Consolare Valeria 1, 98123 Messina, ME, Italy

**Keywords:** 3D printing, guided bone regeneration, GBR, oral, mesh, membrane, bone substitutes, implant, abutment, dentistry, implantology

## Abstract

**Background:** The new frontiers of computer-based surgery, technology, and material advances, have allowed for customized 3D printed manufacturing to become widespread in guided bone regeneration (GBR) in oral implantology. The shape, structural, mechanical, and biological manufacturing characteristics achieved through 3D printing technologies allow for the customization of implant-prosthetic rehabilitations and GBR procedures according to patient-specific needs, reducing complications and surgery time. Therefore, the present narrative review aims to elucidate the 3D-printing digital radiographic process, materials, indications, 3D printed manufacturing-controlled characteristics, histological findings, complications, patient-reported outcomes, and short- and long-term clinical considerations of customized 3D printed mesh, membranes, bone substitutes, and dental implants applied to GBR in oral implantology. **Methods:** An electronic search was performed through MEDLINE/PubMed, Scopus, BioMed Central, and Web of Science until 30 June 2024. **Results:** Three-dimensionally printed titanium meshes and bone substitutes registered successful outcomes in vertical/horizontal bone defect regeneration. Three-dimensionally printed polymeric membranes could link the advantages of conventional resorbable and non-resorbable membranes. Few data on customized 3D printed dental implants and abutments are available, but in vitro and animal studies have shown new promising designs that could improve their mechanical properties and tribocorrosion-associated complications. **Conclusions:** While 3D printing technology has demonstrated potential in GBR, additional human studies are needed to evaluate the short- and long-term follow-up of peri-implant bone levels and volumes following prosthetic functional loading.

## 1. Introduction

In recent years, guided surgery, also called computer-based or template-assisted surgery, has become popular in oral surgery for its reported advantages, such as lower post-operative swelling and pain, as well as less marginal bone loss after computed-based implant placement [1]. Several advantages have been reported, even if a digital software learning curve and project manufacturers are required [2,3,4].

Based on new knowledge, technologies such as three-dimensional (3D) printing, and materials available, understanding the new frontiers of computer-based surgery and its applications in customized 3D printed guided bone regeneration (GBR) in oral implantology is crucial for customizing implant-prosthetic rehabilitations according to patient-specific characteristics and needs, achieves high rates of functional and esthetic success, reduces the intra-operative duration of surgery, as well as the risk of complications, by starting from accurate pre-operative planning favored by the use of the technology itself.

Therefore, the present narrative review aims to elucidate the digital radiographic process in the 3D printing workflow, materials, clinical indications, 3D printed manufacturing-controlled characteristics, histological findings of healing processes, possible complications and their management, patient-reported outcomes, and clinical considerations at short- and long-term follow-up of available customized mesh, membranes, bone substitutes, and 3D printed dental implants applied to GBR in oral implantology.

GBR surgery is one of the most common surgical techniques used to regenerate the alveolar ridge bone undergoing biological bone changes, absorption, and atrophy after tooth loss [5]. Several studies have reported short- and long-term success after GBR surgery in achieving vertical and horizontal alveolar ridge bone augmentation [6]. GBR surgery allows the placement of dental implants, which are widely used for edentulism rehabilitation and require adequate quality and quantity of bone to achieve function and esthetic success [6].

According to the Consensus Report of the Guided Bone Regeneration Symposium in Bologna (2016), GBR was considered a bone reconstruction technique that used non-resorbable or resorbable membranes, titanium meshes, or plates covered with membranes of collagen or synthetic polymers [6]. No surgical technique involving sinus lift, onlay, or inlay graft placement was considered a GBR technique [6].

The biological rationale of the GBR therapeutic concept was proposed for the first time by Dahlin et al. [7] in 1988, and it is based on the achievement of an isolated anatomical site using occlusive barriers to obtain the mechanical exclusion of undesirable soft tissue and to induce osteogenic cell growth in the bone defect [6]. The barriers, such as mesh and membrane, have the role of excluding non-osteogenic cells and creating and maintaining the space [6] to ensure adequate support for the bone graft materials [5]. In the management of severe vertical and/or horizontal bone defects, the use of mesh, which shows greater osteogenic proprieties and mechanical performance than membranes, is recommended [5].

Bone graft materials placed in the bone defects should be used as scaffolds to guide osteogenic cells to form the new bone. If the bone graft materials lack support, they can be displaced by local stress forces, and bone regeneration may be compromised [5].

Recent clinical studies focused attention on the advantages of customized 3D printed mesh, membrane, bone graft, and dental implant, using the newly available technologies spreading in the new digital era [2]. In the 15th European Workshop on Periodontology on Bone Regeneration (2019), the manufacturing of customized biomaterials from 3D patient data was identified as the future of craniomaxillofacial bone regeneration [8].

## 2. Digital Radiographic Process in 3D Printing Workflow

In a fully digital approach, the workflow includes three steps: image acquisition, post-processing, and 3D printing [9].

In the image acquisition step, virtual surgical planning (VSP) is a useful pre-operative tool that requires the acquisition of patient-specific data for 3D digital (or digitalized) study models and images through an intraoral scan, computed tomography (CT), or cone beam computed tomography (CBCT) [1,10].

In the post-processing step, the standard triangulated language (STL) of diagnostic wax-up will be matched with the CBCT DICOM file, generating digital data compatible with a 3D manufacturing machine.

The patient’s bone defect will be reconstructed with computer-aided manufacturing and design (CAD/CAM) technology to generate the necessary instructions for the 3D manufacturing machines [2,11].

In this phase, the customized mesh, membrane, bone graft, and/or dental implant will be designed by taking into account the shape, geometry, and volume of the bone defect, as well as the ideal final dental implant-supported prosthesis position using 3D software [12].

The surgeon can digitally manipulate the 3D models for a careful patient-specific assessment and planning in the pre-operative phase. The VSP supports allow for both the visualization and the surgery simulation, promoting intra-operative precision, predictability, and customization [10]. The pre-operative 3D digital or digitalized study models allow for the combination of patient-specific titanium mesh, membranes, bone graft, or dental implants, as well as for prosthetically guided regeneration (PGR) [1]. The overlaying of a digital wax-up can make the bone regeneration prosthetically guided, and according to a fully digital workflow, it can also include customized CAD/CAM prosthetic restoration [1,2]. Therefore, the 3D models can be printed to analyze and simulate the analogic models of the bone defect and surgery [2].

Moreover, Kim et al. [10] reported the efficacy of VSP in the management of patient expectations, which can promote satisfaction by improving patient–surgeon communications due to the opportunity to provide illustrative outputs to patients, which makes them aware of the surgical process and final results.

The manufacturers design the models in the 3D software, and the obtained file will be converted into a printer-readable file [13].

The 3D printing phase, also known as additive manufacturing, is applied for several dental treatments; in fact, prosthetic bridges and crowns, custom models, surgical guides and tools, orthodontic aligners, and orthodontic braces have been fabricated [14].

The American Society of Testing and Materials defined additive manufacturing as “a process of joining materials to make objects from 3D model data, usually layer upon layer, as opposed to subtractive manufacturing methodologies” [15]. The 3D-printed products in dentistry have usually been created using layer-by-layer approaches based on a CAD model. Stereolithography, selective laser sintering and melting, direct metal laser sintering, electron beam melting, and fused deposit modeling are examples of additive manufacturing techniques [14].

The post-printing stage can require a spinning and microscopical assessment of the printed product [16]. The metallic residues and surface topology after 3D printing should be eliminated through post-processing techniques to minimize inflammatory, reactive, and hypersensitivity reactions [17].

The aforementioned CAD/CAM products’ production process can be categorized as chairside, laboratory, or centralized [18].

Chairside production does not require the involvement of any laboratory; in fact, dentists take an impression chairside and produce the manufactured product. This approach has the advantage of a short time required to produce the manufactured product, which is fabricated based on the same appointment as the image acquisition. However, it has the limitations of higher economic costs [18].

Laboratory production requires the involvement of a laboratory, which fabricates the product based on the dentist’s image acquisition [18].

Centralized production requires that the dentist take the impression that the laboratory digitized as the master cast and send it to an outsourced laboratory that will fabricate the product and send it back to the dentist [18].

The sterilization of 3D printed products is required after the post-printing stage. The sterilization of 3D printed medical devices is a common concern, particularly for devices used for implantation and not in metal due to the possible changes that processes with heat or physical/chemical agents may induce in certain polymers [19]. The 3D printed medical devices used in GBR surgery, which are implanted, should be sterile when implanted to reduce the risk of infection and post-operative complications and to increase the surgery’s success [20]. The guidelines for the sterilization of medical devices commercially available are defined by the manufacturers based on industrial tests of approved protocols of sterilization, while the sterilization of 3D printed medical devices by surgical units is a gray area without guidelines, standards, or regulation [21]. However, most materials used by 3D printing machines are compatible with the sterilization methods for surgical instruments recognized by the Center for Disease Control, such as thermal sterilization by steam or dry heat (also known as autoclave or moist heat sterilization) or low-temperature methods such as chemical (e.g., hydrogen peroxide, ethylene oxide, and peracetic acid) or radiation methods (e.g., ultraviolet or ionizing radiation) [19,22].

However, low-temperature sterilization requires much time and is often not affordable to many hospitals or private dental settings [23].

Heat-dependent sterilization is the most commonly used in dental settings, and it is non-toxic and safe for the environment. This method requires high heat for a long time, which should not be eligible for materials sensitive to temperature, particularly if several repeated sterilizations are required [21]. However, one sterilization is necessary for medical devices used for implantation as in GBR surgery, and even if some polymers, such as polylactic acid, used for the 3D printing of membranes are thermoplastic, the polymers have a high point of melting (250 °C for polylactic acid) and can be subjected to steam sterilization at 121 °C [19] without significantly changing the morphology by conventional autoclaving [23]. Polyglycolic acid and polycaprolactone are other suitable materials [21].

Radiation sterilization is not suitable in the hospital or private dental setting but is mainly used in the medical device industry [22]. Furthermore, a negative impact on the polymer properties has been recognized [20].

The chemical sterilization of 3D printed medical devices with ethylene oxide or peracetic acid should be avoided [21,22]. The first causes toxic products and changes the structures of several polymers like polylactic acid [22]. The second can potentially change the biochemical and structural properties of the polymers [22].

Unlike hydrogen peroxide, low-temperature sterilization is safe, effective [22], and suitable for heat-sensitive materials like polylactic acid [21]. Furthermore, this method of sterilization requires a short time (from 28 to 74 min), but the main disadvantage is the difficulty in the real-time hydrogen peroxide concentration [21].

**Figure 1:**
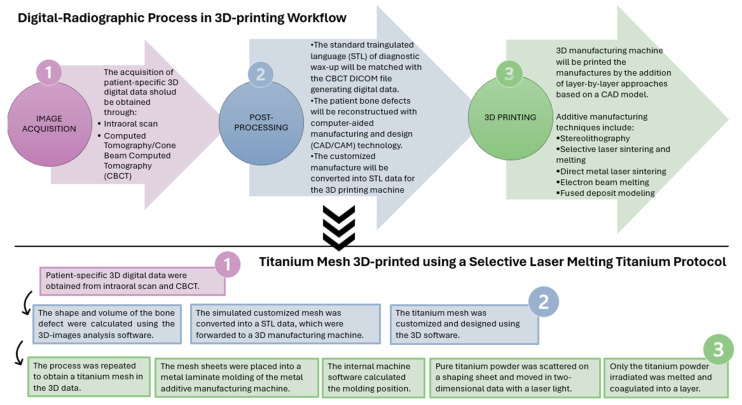
The three steps (image acquisition, post-processing, 3D printing) of the digital radiographic process in the 3D printing workflow, and the specific case of a titanium mesh 3D printed using a selective laser melting titanium protocol.

Figure 1 summarizes the digital radiographic process in the 3D printing workflow and the specific case of a customized titanium mesh fabricated using a selective laser melting titanium protocol [24].

## 3. Materials and Methods

### 3.1. Search Strategy

An electronic search was started on 13 May 2024 and was carried out on the MEDLINE/PubMed, Scopus, BioMed Central, and Web of Science databases to retrieve case reports; case series; case–control, cross-sectional, and randomized clinical trials; retrospective and prospective studies; and in vitro and animal studies published in the English language. The search was updated and concluded on the same databases and with the same search strategy on 30 June 2024.

In combination with Boolean operators, the following keywords were used for the electronic search: (3D-printed OR customized OR three-dimensional printed) AND (guided bone regeneration OR GBR OR customized bone regeneration) AND (mesh OR membrane OR bone substitute OR implant OR abutment) AND (oral OR dentistry OR implantology).

The filters utilized in all the databases were the English language and published articles. No restriction on the date of publication was applied.

An additional manual search was carried out by screening the lists of references of studies included via an electronic search to retrieve additional potentially relevant records.

The Mendeley Reference Manager tool (version 2.80.1) was used to manage references.

### 3.2. Study Selection and Eligibility Criteria

The results from the electronic research in the databases were collected, the records were screened, and the duplicates were eliminated, while the records not relevant to the main topic of the present narrative review were considered not eligible and were eliminated. The full texts of the remaining potentially relevant or ambiguous records were reviewed. The records not compliant with the inclusion/exclusion criteria were excluded, while the other articles were included in the present narrative review.

The inclusion criteria were as follows: case reports; case series; case–control, cross-sectional, and randomized clinical trials; retrospective and prospective studies; and in vitro and animal studies published in the English language concerning at least one aspect from among the materials, clinical indications, 3D printed manufacturing-controlled characteristics, histological findings of healing processes, possible complications and their management, patient-reported outcomes, and clinical outcomes at short- and long-term follow-up of at least one 3D printed customized product applied to GBR in oral implantology (mesh, membranes, bone substitutes, and endosseous dental implants).

The exclusion criteria were as follows: narrative reviews, scoping reviews, and systematic reviews; articles not published (e.g., in press or accepted); not in the English language; or articles whose main topic did not concern at least one aspect among the materials, clinical indications, 3D printed manufacturing-controlled characteristics, histological findings of healing processes, possible complications and their management, patient-reported outcomes, and clinical outcomes at short- and long-term follow-up of almost one 3D printed customized product applied to GBR in oral implantology (mesh, membranes, bone substitutes, and endosseous dental implants).

**Figure 2:**
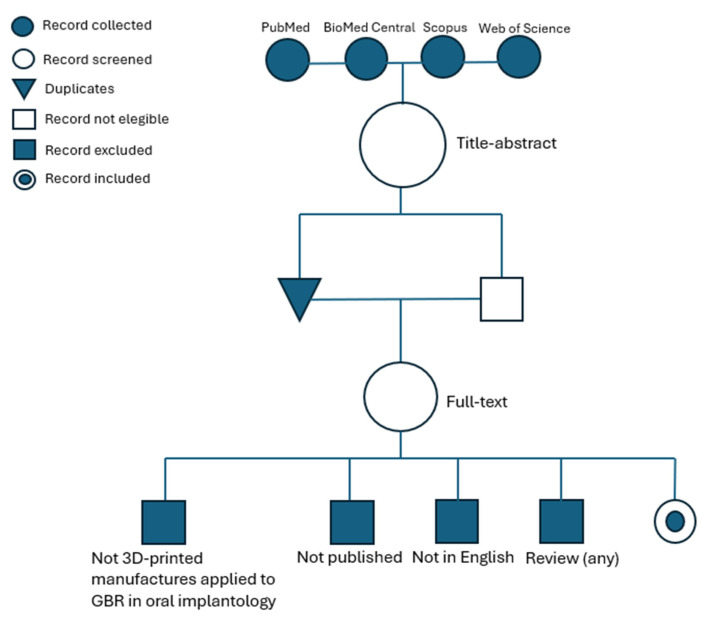
Flowchart of the study selection process from the records collected by electronic search to the records included after the exclusion of duplicates, records not eligible, and records not compliant with the inclusion/exclusion criteria.

Figure 2 summarizes the study selection process through a flowchart.

### 3.3. Data Extraction, Collection, and Synthesis

Data from the included studies were extracted and collected on a dedicated extraction form using Microsoft Excel software 2019 (Microsoft Corporation, Redmond, WA, USA).

As for the extraction and collection process, a narrative qualitative synthesis was carried out separately for the different 3D printed customized manufacturers applied to GBR in oral implantology: mesh, membranes, bone substitutes, and dental implants. Furthermore, for each different 3D printed customized manufacturer, a qualitative synthesis was performed focusing on the materials, clinical indications, 3D printed manufacturing-controlled characteristics, histological findings of healing processes, possible complications and their management, patient-reported outcomes, and clinical considerations at short- and long-term follow-up.

If any studies registered data for several 3D printed products, the study was considered separately for each product type, and the data were extracted and collected separately according to the type of product.

## 4. Mesh

In total, data from fourteen human studies [6,19,20,21,22,23,24,25,26,27,28,29,30,31], in which customized 3D printed meshes were manufactured and applied in GRB in oral implantology surgery, were extracted and collected (Table 1).

In total, 184 customized 3D printed meshes were reported in the studies included in the present narrative review.

### 4.1. Material and Clinical Indications

Customized titanium meshes were developed to facilitate the fitting of titanium mesh with the specific patient bone defect shape [24] and to obtain pre-determined width and thickness characteristics [2]. Customized meshes showed several advantages, such as quicker surgery, personalized fitting, reduction in the pin to obtain mesh retention, smoother edges, less mucosal stress, and the possibility to fill the 3D printed mesh with bone graft outside the oral cavity, reducing the risk of graft intraoral dissemination or contamination [2,26].

Customized titanium meshes were the most utilized in GBR [2,11].

Other materials, broadly applied in orthopedics, have been used in oral surgery in recent years. Customized poly-ether-ether-keton (PEEK) meshes have high biocompatibility and inertness [2]. PEEK is a semi-crystalline polyaromatic and thermoplastic polymer that has shown predictable outcomes in the reconstruction of maxillofacial defects [33].

Zirconia is a polycrystalline ceramic that is considered an innovative and alternative material for 3D printed customized mesh fabrication due to its high biocompatibility, scarce ability to tissue integration, and less biofilm adhesion and inflammatory response than titanium mesh [13].

### 4.2. Three-Dimensionally Printed Manufacturing-Controlled Characteristics

Customized 3D printed meshes should be designed, tailored to both the mesh macro- and micro-structure.

The appropriate thickness of a 3D printed titanium mesh must guarantee resistance to deformation under the stress forces during the healing period and rigidity to maintain space for bone regeneration, but at the same time, it must not adversely influence soft tissue healing. In laboratory tests, titanium meshes with 0.4 mm thickness showed good response in both strength and the stimulation of the mucosa [37]. Previous studies evaluated the mechanical proprieties of 3D printed meshes with different pore sizes and thicknesses [37]. The mechanical tests showed that 3D printed titanium meshes with 0.3 mm in thickness were not safe for GBR surgery due to the high fracture rates, while meshes with 0.4–0.5 mm in thickness should be used [37].

However, the thickness of the customized titanium mesh should be defined clinically; in fact, for the GBR of the anterior area with a single tooth missing, a customized titanium mesh of 0.3 mm in thickness, has been indicated for in patients with thin mucosa to reduce the risk of mesh exposure [37]. In contrast, in the posterior area with multiple teeth missing, a thicker customized titanium mesh is preferred [37].

Concerning the effect of titanium meshes’ pore size on the osteogenesis proprieties, the results have been controversial. Some studies reported that macroporous (millimeter range) meshes favor bone regeneration compared to microporous (micron range) meshes [38]. However, other studies have reported the advantage of microporous over microporous titanium mesh in GBR [39].

Porous networks of 3D printed titanium–niobium (Ti-Nb) alloy meshes have also been developed. A porous coating of gelatin/doxycycline/chitosan was created on the meshes. Ti-Nb meshes with porous coating have been shown to be effective in space-maintaining, fibroblast cell growth prevention, and inhibiting bacterial colonization [40].

Further studies should identify the mesh pore size that can inhibit soft tissue overgrowth and facilitate bone regeneration in GBR surgery.

Finally, it must be considered that the mechanical properties of customized and conventional titanium meshes weaken as the pore diameter increases. In titanium mesh of 3–5 mm in diameter, a higher range of macropores had a minor influence on the mesh’s mechanical properties [37].

### 4.3. Histological Findings of Healing Processes

Twelve [1,25,26,27,28,29,30,31,32,33,34,35] of the included studies reported the histological findings of the healing process after GBR surgery using a customized 3D printed mesh. The findings of histological evaluations ranged from 6 months [25,27,28,30,32,33,34,35] to 12 months [1].

Dellavia et al. [29] assessed the histological features of a bone sample biopsy collected after 9 months of GBR during the removal of 3D printed titanium mesh.

In all twenty patients, 3D printed titanium meshes were not associated with histological signs of adverse reactions, necrosis, fibrosis, or ectopic adipose tissue. The bone was in close contact with the titanium mesh, well organized, and mineralized at different stages; the medullary spaces were characterized by blast-like cells, blood vessels, and rare inflammatory cells. The bone margins showed remodeling fronts with diffused osteoid cells.

In the three patients with 3D printed mesh exposure, the oral mucosa surrounding the mesh was characterized by the compresence of bone materials and dense connective tissue with numerous fibroblast-like cells [29].

Other studies have also reported the presence of a thin (1–2 mm) layer of soft tissue, defined as “pseudo-periosteum”, upon bone regeneration through GBR surgery with titanium mesh [30].

Morphological assessments and the morphometric data have indicated that the 3D printed titanium meshes induce bone tissue regeneration that was well organized and structured, vital, and with active turnover [26,29].

Notably, the histological findings reported in cases of delayed mesh exposures seem to not significantly influence the healing process or affect the histologically regenerated bone tissues [29].

The aforementioned main histological findings from GBR surgery with 3D printed titanium mesh are aligned with data from previous human studies using traditional titanium meshes [41]. Also, the proportion of bone regenerated tissue and residual graft recorded at 9 months after GBR were comparable to those of traditional titanium meshes [42].

### 4.4. Complications and Their Management

The main reported complications during the healing process after surgery with customized 3D printed meshes were wound dehiscence and mesh exposure (reported in 40 cases of the 184 3D printed mesh included in the present narrative review), which can be classified as early exposure if it occurred within 4 weeks after surgery and delayed exposure if it occurred after 4 weeks [2].

Early exposure is associated with a reduction in bone regeneration and an increase in fibrous tissue. Management requires the application of chlorhexidine (0.2% gels/mouthwashes/spray) and the curettage of the interested site. The exposed mesh should be removed, disinfection treatment should be carried out, and topical antibiotics should be applied when a graft infection is suspected [2,43]. In the present study, 25 cases [26,27,28,29,32,33] of early mesh exposure occurred, and in 2 cases, the meshes had to be removed [26].

Delayed exposure could induce the resorption of graft substitutes (reported in nine cases [26,32] in the present narrative review). However, the delayed exposure mesh can be left in situ, and the treatment comprises plaque management, disinfection with chlorhexidine, and the smoothing of the mesh with carbide or diamond burs [2,44,45].

Three-dimensionally printed mesh exposures ranged from 22.6% to 33% [11,26,46,47]. Despite the reported advantage of 3D printed mesh in reducing soft tissue tension, the titanium mesh exposure rate seems to not be influenced by the mesh type (conventional or 3D printed), bone substitute material, or associated absorbable membranes [48].

However, in spite of the high percentage of mesh exposure, bone regeneration can be obtained, and an early re-epithelization surrounding the exposed mesh occurs, creating a natural barrier for the grafts [26].

Concerning the 3D printed customized zirconia, the study of Mandelli et al. [13] showed that no infections were recorded following exposures of zirconia meshes.

Specifically for zirconia mesh, the fixation with screws should not be carried out by applying excessive forces during tightening, which can lead to zirconia fracture due to the brittleness and low elastic modulus of the material [13].

### 4.5. Clinical Considerations

Several studies have concluded that customized 3D printed titanium meshes can be considered a successful solution in GBR surgery for horizontal and vertical bone augmentation [1,24,25,26,28,30,31,32,33,34,35] and also to regenerate complex bone defects in the esthetic area [31].

Customized 3D printed titanium meshes in GBR procedures have registered relevant bone augmentation in width (up to 11.48 mm, with a mean of 6.35 mm) and in height (up to 8.90 mm, with a mean of 4.78 mm) [26].

The use of a 3D printed mesh with or without a membrane has not registered significant statistical differences, even if the placement of a membrane seems to reduce the healing complication rates [28].

Conventional titanium meshes are generally rigid and sheet-like, and their cutting or refinishing is often necessary during surgical procedures, which results in higher operation times compared to customized 3D printed meshes [36]. Instead, no cases of the necessity of intra-operative modification of customized 3D printed meshes have been reported [26].

Instead, considering the patient’s point of view, only one study regarding customized 3D printed mesh that evaluated patient-reported outcomes was retrieved by the electronic search in the present narrative review. Navarro Cuéllar et al. [34] reported that six patients considered the final result of GRB surgery as “good aesthetic”, created using a customized 3D printed titanium mesh. The remaining two patients of study [34] said the final results were “fair”. Unfortunately, no patient-reported outcomes concerning the patients’ pain, swelling, or difficulty in opening the mouth after GBR surgery with customized 3D printed titanium meshes were found. Furthermore, no patient-reported outcomes on other non-titanium meshes were reported precluding the possibility of comparing the different materials 3D printed meshes based on the patient’s reported outcomes.

These data could influence the decision-making process and provide adequate information to patients on the risk factors, recovery after GBR surgery, and post-operative experience [49].

Concerning the differences between customized 3D printed titanium and PEEK meshes, in the randomized clinical trial of Mounir et al. [33], no significant differences were found in the 3D bone gain between the control group in which the maxillary alveolar defects were treated with bone graft loaded in a prebent titanium mesh or a customized PEEK mesh.

Specifically for zirconia mesh, the possibility of 3D printing has surpassed the zirconia limitation of inflexibility, which makes it impossible to adapt to the bone defect shape if not 3D printed [13].

Furthermore, the 3D printed customized zirconia meshes showed their safety and predictability in GBR surgery in humans, even if the studies on 3D printed zirconia meshes were limited and long-term follow-ups were not available [13]. An advantage of zirconia mesh over the other mesh is the absence of zirconia mesh adhesion and integration with the surrounding hard and soft tissues, which reduces the time of re-opening surgery for mesh removal [13].

Despite these advantages of customized 3D printed meshes, it may be considered that further studies should investigate the short- and long-term follow-up of peri-implant bone levels and volumes after the prosthetic functional loading, as they are necessary to fully evaluate the potential advantages of 3D printed meshes over time.

## 5. Membranes

In total, data from seven animal studies [50,51,52,53,54,55,56], one human/ex vivo study [57], and one in vitro study [58], in which customized 3D printed membranes were manufactured and applied in GRB in oral implantology surgery, were extracted and collected (Table 2).

### 5.1. Materials and Clinical Indications

In the customization of 3D printed membranes, the possibility of controlling the sizes and geometry of the manufacturers, and the reduction in material waste are the main advantages of the 3D printing techniques [60].

Customized 3D printed membranes fabricated with biocompatible polymers are characterized by strong mechanical properties and biodegradation, which eliminate the need for non-resorbable membrane removal associated with additional surgical trauma [10].

Three-dimensionally printed polymers include the polyesters of hydroxycarboxylic acid as synthetic polyglycolide, polylactide, poly-3-hydroxybutyrate, and their copolymers [53].

Polylactic acid (PLA) is a 100% biodegradable polymer, which, compared to other materials, such as acrylonitrile butadiene styrene, is easier to print and less toxic due to a reduced styrene production. Styrene is a possible carcinogen but is less strong [57].

PLA has shown a mean resorption time in vivo of 1–2 years [51,56].

Polyglycolic acid (PGA) is a rapid biodegradable polymer with a resorption time of up to six months, unlike PLA. The strength properties of membranes decrease with increasing resorption rates over time. Hence, 3D printed pure PGA membranes are not suitable for GRB procedures [53].

PGA and PLA biodegradation occurs through hydrolysis and macrophage cell response [53]. PLA/PGA copolymers have shown the ability to modify the mechanical membrane properties and the biodegradation times up to 18 months [53].

Polycaprolactone (PCL) is a biodegradable polymer with a slower degradation rate, which can be more than 12 months, due to its hydrophobicity [59]. PCL has less cell affinity compared to PLGA but excellent mechanical properties [52]. PCL/PLGA blended for membrane printing has shown mechanical and biological membrane advantages due to the complement weaknesses of the two materials [52].

PCL and PCL/PLGA membranes were 3D printed even with the addition of the β-tricalcium phosphate (β-TCP) [52,59], a biodegradable, hydrophilic, and low-ductile/flexible bio-ceramic material with several advantages such as bioactivity, biocompatibility, and osteoconductive properties. The addition of β-TCP in the membrane fabrications in synthetic polymers allows the enhancement of the low bioactivity of polymers [16], due to the β-TCP’s ability to release ions of calcium, inducing bone regeneration, and the elastic modulus and surface roughness registered increased [52].

### 5.2. Three-Dimensionally Printed Manufacturing-Controlled Characteristics

The mechanical and biological properties of 3D printed membranes should be controlled during manufacturing, using the polymers alone or in combination, modifying the membrane’s shape, pore size, thickness, and biodegradable rate [56].

The membrane pore size and the interconnecting structures play an important role in the transport of proteins and growth factors, as well as in the migration of osteogenic and angiogenic cells [56].

However, the optimum pore size able to prevent soft tissue ingrowth and facilitate bone and vessel regeneration has still not been defined [56].

A microporous membrane should prevent the ingrowth of soft tissue, reduce bacterial penetration, ensure a large surface area for the attachment of cells, and reduce tissue integration, allowing for a simple second surgery for the membrane’s removal. Higher strength and elastic moduli, and consequently better space maintenance properties but minor manageability, have been reported [58].

In contrast, macroporous membranes should guarantee stability through tissue integration, but consequently increased difficulty for membrane removal, good osteogenic and angiogenic cell penetrations, and flexibility [58].

Lundgren et al. [61] reported that pore sizes ranging from 25 to 300 µm would be optimal for bone regeneration under the membranes. Other studies [62,63] showed that pore sizes of 100 µm were the smallest for enhancing the osteogenic and angiogenic cell migration, while the pore sizes should be greater than 150 µm to facilitate new bone regeneration [38]. A larger pore size allowed fibroblast overgrowth in the bone defect, inhibiting the osteoblasts’ growth [54].

In contrast, a totally occlusive membrane delayed bone regeneration due to the impossibility of the migration of angiogenic cells, which led to avascular regeneration [56].

The membrane degradation rate is another important property linked to cell vitality and growth. Membranes should degrade slowly during the initial bone growth to maintain the space and exclude nonosteogenic cells, and subsequently more rapidly to provide the space for bone regeneration [59].

The ratio of PLA and PGA in the manufacturing of polylactic-co-glycolic acid (PLGA) membranes plays an important role in conditioning the biodegradable rates [56].

In the in vitro and animal study on rats by Petposri et al. [56], 3D printed customized membranes in PLGA with different LA:GA ratios were compared (group A, LA:GA = 70:30; group B, LA:GA = 10:90). The results concerning the biodegradability rate showed that PLGA (10:90) had a significantly higher rapid rate of degradation than the PLGA (70:30) membranes and lost stability proprieties after 15 days. In addition, the PLGA (10:90) released more acid due to the degradation of glycolide and lactide polymers, which created an environment incompatible with cell viability [56].

This study demonstrated that 3D printed customized PLGA (70:30) membranes were suitable for GBR surgery, in contrast to PLGA (10:90) membranes.

The membrane elongation at break and tensile strength should be maximized to optimize the membrane deformation properties and load capacity before breakage [58]. Furthermore, the elastic modulus should not be high to prevent soft tissue damage [64] but neither low so as not to guarantee space maintenance [58].

The bioresorbable membranes commercially available have elastic moduli from 30.6 to 700 MPa, and tensile strengths from 3.5 to 22.5 MPa [58]. Considering that the lack of space maintenance is the major disadvantage of bioresorbable membranes, Zhang et al. [58] suggested that using 3D printing technology to create novel membranes with slightly higher elastic moduli should be ideal. Comparing the mechanical properties of different 3D printed PLA membranes with variable pore sizes, Zhang et al. [58] registered the highest elastic modulus, strength, and low elongation at break for the no-pore membranes; the small-pore membranes showed a moderate elastic modulus and strength, and a low elongation to break; while the large-pore membranes had better ductility, but with the lowest strength and elastic modulus. This in vitro study demonstrated that the strength of the membrane decreases as pore size increases [58]. The authors concluded that membranes with different pore sizes can be required in specific clinical conditions [58]. In particular, if stronger support is required, as in the case of large bone augmentation or defects, the no-pore membrane can be considered [58]. However, as mentioned above, membranes without pores delay bone regeneration and hinder the migration of angiogenic cells [56]; if better manageability is required, as in small bone defects, or if there is the support of bone substitutes, the large-pore membrane should be preferred; finally, if moderate property is required, the small membrane should be taken into account [58].

### 5.3. Histological Findings of Healing Processes

Six [50,52,53,54,55,56] of the included studies reported the histological findings of the healing process after GBR surgery performed using a customized 3D printed membrane. The findings of histological evaluations reported always involved animal models, and the reported follow-up ranged from 2 days [55] to 8 weeks [52,55,56,59].

Interestingly, during the first two weeks after GBR surgery with 3D printed customized PLGA (30:70) membranes, chronic inflammatory and giant cells were detected due to membrane degradation kinetics.

In addition, the PLGA (30:70) membranes, as a consequence of the water uptake during hydrolysis, were slightly swollen [56].

However, the same histological finding was recorded during the healing process of GBR surgery with collagen membranes, which were degraded by macrophages and polymorphonuclear cells [56].

The regenerated new bone using the 3D printed PLGA (30:70) membranes was detected mainly at the peripheries of the host bone, but new bone and new blood vessels were also found under the PLGA membranes. These findings may be linked to the osteoconductive properties of PLGA membranes [56].

Instead, in considering the influence of membrane pore size in the healing process, in the in vitro study of Zhang et al. [58], which compared 3D printed PLA membranes with different pore sizes, the SEM images showed that cell proliferation increased with the decrease in pore sizes at all time points, even if significant differences were reported only between one and three days [58]. This finding is in accordance with the study of Marouf et al. [65], which investigated the effectiveness of high-density expanded polytetrafluoroethylene (PTFE) membranes and of semipermeable expanded PTFE membranes in rabbit animal models. Both for 3D printed membranes and conventional PTFE membranes, it was hypothesized that the microporous membrane slowed the vascular ingrowth and did not allow adequate nutrient supply over time in the bone defect, even if in the early stages, the membrane area in contact with osteogenic cells was higher than the contact area of a large-size porous membrane [65].

Regarding the healing process of PCL membranes, in the study of Shim et al. [59], the newly regenerated bone in a beagle dogs’ buccal defects was attached under the PCL and PCL/β-TCP membrane, while in the control group (collagen membranes) the membranes floated away, and graft materials were scattered from the buccal defects.

Furthermore, the study of Lee et al. [55] showed good PCL/β-TCP membrane biocompatibility and the absence of inflammatory cells for up to eight weeks.

### 5.4. Complications and Their Management

The main complication reported in the included studies on 3D printed membranes was PLGA degradation, which generated acidic products that caused inflammatory response and rapid swelling in the in vivo animal model [56,59].

However, 3D printing technology is itself a means to avoid this complication; in fact, as reported by the authors of [56], the toxic products and the progressive lack of mechanical properties caused by the PLGA degradation can be managed by controlling the ratio between PLA and PGA [56,59].

In contrast to 3D printed mesh, data retrieved from human studies concerning 3D printed membranes are still poor, and current knowledge about the 3D printed membranes complications does not yet allow them to be compared with conventional membranes.

### 5.5. Clinical Considerations

According to Scantlebury et al. [66], the membranes used in tissue regeneration surgery must fulfill five main criteria: (i.) tissue integration; (ii.) cell occlusivity; (iii.) clinical manageability; (iv.) space making; and (v.) biocompatibility. These properties are determined by the composition of membrane material, physicochemical characteristics, and structure [58].

Resorbable membranes, like collagen membranes, have been preferred to non-resorbable ones because the mechanical and space maintenance properties of resorbable membranes decrease in parallel with the resorbable process, and this can be a limitation when the membrane loses its mechanical property before bone healing [55]. However, resorbable membranes do not require reintervention surgery for membrane removal, which can result in further patient discomfort, treatment duration, risk of tissue damage, and additional economic costs [52]. The study of Kofina et al. [67] reported that the post-operative symptoms after GBR with conventional resorbable membranes and particulate bone substitutes worsened two days after the surgery and the oral health-related quality of life was significantly impacted by swelling, pain, surgery duration, difficulty in opening the mouth, and flap advancement. Considering the reported advantage of 3D printed membranes in reducing the duration of GBR surgery, a comparison between the patient-reported outcomes after GBR surgery with traditional membranes and 3D printed membranes might be interesting in evaluating real-patient feedback. Unfortunately, no patient-reported outcomes have been reported in the included human studies that used customized 3D printed membranes for GBR surgery. Further studies may be necessary to investigate whether customized membranes can improve patient-reported outcomes after surgery.

In contrast, non-resorbable membranes in expanded-polytetrafluoroethylene (e-PTFE) or dense-PTFE (d-PTFE) have been preferred for the reconstruction of large bone defects due to their superior structural stability as well as space maintenance properties [55].

However, non-resorbable membranes are associated with frequent membrane exposure, risk of infections, morbidity, and wound dehiscence [55].

Therefore, the manufacturing of new membranes utilizing 3D printed technology was proposed to link the biodegradability, tissue integration ability, and biocompatibility of the resorbable membranes, with superior mechanical stability, longevity, and space maintenance properties of the non-resorbable membranes [52,55].

Bioresorbable synthetic polymers were used for the manufacturing of scaffolds in tissue engineering through electrospinning, foaming/particulate leaching, freeze-drying, and phase separation [52]. However, these conventional techniques of fabrication require toxic solvents, which have adverse effects on human cells if not completely cleared [52]. Three-dimensional printing technology does not require these solvents, and the manufacturing-controlling membrane characteristics, such as thickness, shape, or pore size, can be controlled and handled more easily than conventional technologies [52].

Collagen membranes are biocompatible and biodegradable, have low immunogenicity, and are easy to manage and adapt to tissue [52]. In GBR surgery, collagen membranes are considered the gold standard [59].

However, the collagen membranes have a mean resorption time ranging from 5 to 28 days, with complete resorption after 8 weeks, which can limit the soft tissue down growth in the early soft tissue healing process, but the mechanical strength and space maintenance proprieties decrease during the longer bone healing process [52,56].

Petposri et al. [56] demonstrated that eight weeks after GBR surgery, even if the collagen membranes were still detectable, they collapsed into bone defects. Instead, the PLGA (30:70) membranes maintained their shape over the eight weeks [56].

Compared to the collagen membranes, the PCL/β-TCP 3D printed membranes showed similar cell proliferation and osteogenic differentiation but better mechanical properties in GBR procedures performed on buccal mandibular defects from first premolars to the first molars of beagle dogs [59]. Furthermore, PCL and PCL/β-TCP membranes registered similar elastic moduli under wet and dry conditions, while the collagen membranes that absorbed water rapidly decreased. Therefore, PCL and PCL/β-TCP membranes are capable of maintaining their space-making properties also in the presence of blood or saliva due to the PCL hydrophobicity [59]. The progressive biodegradation of 3D printed PCL/β-TCP membranes allow cell growth, inducing bleeding [55].

In addition, after eight weeks from GBR surgery, the group with PCL/β-TCP membranes registered statistically significantly higher values of horizontal width gain of ridge augmentation than both the PCL and the collagen membrane groups, probably due to the osteoconductive propriety of β-TCP [59].

Considering the aforementioned characteristics of the 3D printed PCL/β-TCP membrane, Lee et al. [55] suggested their use as an alternative to collagen membranes in conditions where higher action duration and mechanical properties are required [55].

However, PCL/β-TCP membranes had shown lower wettability compared to collagen membranes. This may represent a possible drawback of PCL/β-TCP membranes for the need for fixation to obtain a stable positioning under a grafted site [55].

Furthermore, the use of commercially available membranes usually requires a pre-treatment by manually bending and trimming the membrane structure to fit it to the bone defect shape [58]. Complete membrane stabilization and tissue integration cannot be guaranteed, and the risk is fibrous connective tissue ingrowth [58].

Therefore, 3D printed customized membranes are promising alternatives to commercially available membranes for the potential possibility of fabricating membranes with different pore sizes based on clinical demand [58].

In addition, drugs and growth factors can be incorporated into 3D printed customized membranes [59].

Finally, novel hybrid membranes were 3D printed, even if their applications have been reported in animal studies for the regeneration of tibia bone defects in canine models [50].

However, early studies on animal models have shown promising results for bone defect regeneration using a 3D printed PCL membrane firmly attached to a layer of gelatin hydrogen [50]. The aim was to generate a membrane with a layer of synthetic polymer and other hydrogel at the interface between soft and hard tissues for GBR surgery. The gelatin matrices helped the cell growth and attachment, while the 3D printed PCL layer ensured the mechanical strength properties of the membrane [50].

In conclusion, most of the studies included in the present narrative review showed superior [50,51,53,56] or comparable [52,59] properties of 3D printed membranes than collagen membranes. However, most of the currently available data on 3D printed membranes resulted from animal studies.

Further studies on humans are needed to compare 3D printed membranes with other traditional membranes.

## 6. Bone Substitutes

In total, data from four human studies [12,68,69,70], one animal study [71], and one in vivo study [72], in which customized 3D printed bone substitutes were manufactured and applied in GRB in oral implantology surgery, were extracted and collected (Table 3).

### 6.1. Materials and Clinical Indications

The bone substitutes could be printed according to the patient-specific defect shape and geometry using 3D printing technology [73], which allows for the customized fabrication of bone substitutes for alveolar ridge augmentation or preservation surgeries (simultaneously, with tooth extraction, the bone defect is filled with a customized 3D printed bone substitute whose size is calculated using software based on CBCT data) [69,70] and also for more extensive surgical mandibular reconstruction [74].

The advantages of printing the bone substitute are its suitability for the defect area and the shortened operation time since there is no need to adapt the bone substitute intra-operatively to the defect shape. Consequently, other advantages of bone substitute 3D printing include customized surface and porous structures, unnecessary autologous bone graft harvest, manual modification resulting in the minimization of contamination risk, and the reduction in bone material waste [70,71]. Moreover, the bone substitute architectural design can imitate both the cortex and trabecular structure of the alveolar bone [12].

Three-dimensionally printed nano-porous hydroxyapatite bone substitutes can be used for ridge preservation procedures due to the materials’ high biocompatibility and scaffold characteristics [69]. Combining a 3D powder printing process with low-temperature phase transformation makes it possible to obtain a low-crystalline nano-hydroxyapatite structure. The bone substitute has both osteoconductivity and osteoclastic resorbability in comparison with the typical high-temperature sintering route [12,68,69,72]. This 3D printed substitute has shown significant resorption reduction and no difference from commercial bone substitutes [69,75].

A 3D printed mixture of hydroxyapatite and β-tricalcium phosphate (60:40 ratio) showed bone regeneration capacity comparable to conventional bone substitute material [70].

Implant stability is not affected by these kinds of materials since the average IST (implant stability test) value did not statistically differ between 3D-printed bone substitute groups and non-3D-printed bone substitute groups [69]. The values reported agree with the ones reported for implants in pristine bone [76].

### 6.2. Three-Dimensionally Printed Manufacturing-Controlled Characteristics

Using specific software, the design of 3D printed substitutes aimed to imitate the alveolar bone structure, taking into account both internal and external structures. The internal trabecular bone could be reproduced, creating an internal porous structure thanks to spherical macropores and cross-combined T-shaped patterns, allowing the reinforcement of this structure to contrast the collapse or damage that can occur during processing [12].

The external structure of the 3D printed bone substitute was produced considering the defect size, which is calculated from the patient CBCT data.

The ideal final dental implant position could also be taken into consideration while creating the contour surface of the substitute, importing the previous data into a dental implant planning software [70]. With a 3D printing machine, the customized bone substitute is built layer-by-layer using the selected material, which can be subsequently dried and sterilized [12].

### 6.3. Histological Findings of Healing Processes

Histological analysis at 5 months after GBR with 3D printed customized bone substitutes showed both newly formed bone and bone substitute particles. These findings agree with the results of conventional bone substitutes [70].

At 6 months, histological analysis showed the interface between the native bone layer and the newly formed bone layer, and the newly formed bone had direct contact with the residual bone substitute, thus indicating osteoconductivity and bioactivity. Histomorphometric analysis revealed connective tissue, with blood vessels being present within it; residual bone substitutes; bone tissue; and newly formed bone tissue. The newly formed bone was present with a statistically significant higher percentage (28.6 ± 1.88%) than connective tissue (20.81 ± 4.41%) and residual bone substitute (19.82 ± 4.07%) [12,69].

No evidence of inflammatory response could be found near the bone substitute material [12,70].

The comparison between alveolar ridge resorption after ridge preservation with 3D printed bone substitute materials and alveolar ridge resorption after ridge preservation with commercial bone substitutes showed no statistically significant differences on a histomorphometric level or concerning 3D changes in soft tissues and bony tissues [69].

### 6.4. Complications and Their Management

The digital radiographic process in the 3D printing workflow requires image data acquisition from a CBCT to identify the defect size and geometry. However, sometimes the accuracy of CBCT cannot be perfectly reliable, and the assessment of shape/geometry bone defects in CBCT could be imprecise. Thus, in some cases, the clinical bone defect shape and geometry might not exactly embrace the 3D printed bone substitutes, due to complex anatomical structure.

The micro-gaps between the customized bone substitute and the bone defect walls can hinder osteogenesis due to the substitute’s micro-movements. Effective design team–clinician communication is essential, and accurate CBCT settings are crucial [70].

Another clinical limitation of 3D printed bone substitutes was reported for the 3D printed ceramic bone substitutes, which, being fragile, had shown fixation challenges. However, given their block-like design, 3D printed bone substitutes should be easily anchored with screws. On the other hand, traditional allogenic bone block substitutes are easy to fix with screws [70]. This limitation of 3D printed bone substitutes highlights the necessity of enhancing their strength.

In the study by Mekcha et al. [12], customized 3D printed nano-hydroxyapatite bone substitutes incorporating concentrated growth factors (CGFs) and covered by platelet-rich fibrin (PRF) membranes were used in GBR surgery, and the outcomes were compared with the group using 3D printed nano-hydroxyapatite bone substitutes without CGFs and PRF. Concerning the oral mucosa dehiscence over 3D printed nanohydroxyapatite bone substitutes without CGFs and PRF, the complications reported by Mekcha et al. [12] within two months of GBR surgery were high (66.67% of patients), resulting in total or partial bone substitute failures. Instead, the 3D printed nanohydroxyapatite bone substitutes combined with CGFs and covered by collagen membranes and also above PRF membranes had shown good soft tissue healing and a lowered failure rate (11.11%) [12].

Therefore, based on the current preliminary results, the authors concluded that GBR surgery using a 3D printed nanohydroxyapatite bone substitute alone is not recommended due to the high risk of complications registered within two months [12]. In contrast, the combined use of CGFs and PRF membranes registered an increased healing index over time significantly higher at 2 months and highest at 6 months compared to the control group [12]. These results should be linked to the PRF’s beneficial effect of accelerating bone regeneration, improving intra-bony defect fill, enhancing bone graft, and so decreasing the occurrence of membrane exposure [12,77,78,79], while CGFs incorporated into the 3D printed bone substitute had enhanced its osteoconductive properties [12,80].

### 6.5. Clinical Considerations

Soft tissue healing can be monitored utilizing a healing index [81]. Mekcha et al. [12] showed a case series of 3D printed nanohydroxyapatite bone block substitutes, and the healing index, while increasing over time, showed a significantly higher score at 2 months (3.75 ± 1.22) and the highest score at 6 months (4.7 ± 0.67). The mean horizontal width at baseline was 4.53 ± 1.80 mm, while at 6 months, it was 7.05 ± 1.34 mm, with a mean bone gain of 2.52 ± 0.54 mm (maximum and minimum bone gains of 3.06 ± 1.02 and 1.69 ± 1.13 mm, respectively) [12].

CBCT radiographs taken at 6 months showed adequate bone volume for implant placement and no significant resorption or non-fixation of the bone substitute [12]. The randomized controlled trial of Kijartorn et al. [69] showed that the average IST value at four months after ridge preservation procedures with 3D printed bone substitutes, thus at the time of implant placement, was adequate for implant osseointegration, indicating good stability. Three months after implant placement, before the insertion of the crown, the average IST value was 73, indicating that good stability can be reached in prosthesis insertion and that the ridge preservation procedure with 3D printed bone substitutes did not compromise the osteointegration [69,76].

In conclusion, customized 3D printed bone substitutes have the advantage of matching the required shape and size of the alveolar bone defects with the graft material and serve as a higher biocompatibility scaffold, thus reducing the operation time by making a gross adjustment of the block substitute to the defect unnecessary.

The use of traditional block-form bone substitutes requires the skills of a surgeon to shape and trim the block to fit the recipient site [12].

Instead, considering the patient’s point of view, few studies have investigated the patient-reported outcomes after GBR surgery with the placement of customized 3D printed bone substitutes [10,12].

Kim et al. [70] suggested that customized 3D printed bone substitutes enhanced patient satisfaction due to the unnecessary need for autogenous bone grafts to be used, thus minimizing the associated patient comorbidities.

Mekcha et al. [12] showed a case series of 12 patients with horizontal ridge defects. These patients underwent augmentation procedures with a 3D printed nonohydroxyapatite block substitute before implant placement. All 12 patients recorded very low pain scores, evaluated with the pain visual analog scale (VAS) ranging from 0 to 10, during surgery (1.41 ± 0.51). The mean scores were lower at 2 weeks (0.92 ± 0.51) and 1 month (0.33 ± 0.49), and no pain in any patients was registered at 2, 3, or 6 months after surgery. No significant differences in pain perceptions were registered at the baseline or 2 weeks after surgery [12].

These findings seem to be comparable with the patient-reported outcomes recorded after GBR surgery with traditional xenograft and/or autogenous bone [49,81]. The average daily pain was reported as mild–moderate for the first 3–4 days after surgery with a gradual decrease to almost no pain in the first week, independently of the bone regeneration surgery type (sinus lift, GBR) or the type of bone substitute (traditional autologous/xenograft, 3D printed) [12,49,82].

## 7. Dental Implant

In total, data from five animal studies [83,84,85,86,87] and from two in vitro studies [88,89], in which customized 3D printed endosseous dental implants were manufactured and applied in GRB in oral implantology surgery, were extracted and collected (Table 4).

### 7.1. Materials and Clinical Indications

Several authors have proposed manufacturing patient-specific implants (PSIs), with or without customized abutments [90], through 3D printing manufacturing machines to design endosseous dental implants that consider the patient defect’s anatomy and respect the implants’ potential fit and the load that will be subjected under the prosthesis [85,88,89,91,92].

Three-dimensionally printed PSIs have shown a high capability of matching the anatomy of the defect without interference, providing improved primary stability, facilitating osseointegration, and reducing the amount of peri-implant bone graft [83,88]. Furthermore, precision PSIs fit in bone defects and should reduce the risk of bacterial implant colonization, minimizing the implant–bone gaps [88].

The use of 3D printed dental implants was also extended to more complex rehabilitation for the reconstruction of a mandibular defect, resulting after a segmental mandibulectomy, which was successfully treated using a traditional dental implant positioned into a 3D printed titanium mandibular implant [93].

Titanium (Ti) and Ti alloys were the materials most commonly used to fabricate traditional dental implants [88] and 3D printed dental implants, for high biocompatibility and mechanical properties [83].

Other materials used for 3D printed dental implants are zirconia, alumina, and PEEK [94].

Zirconia dental implants have shown similar osteointegration rates to titanium dental implants and high biocompatibility [94].

Alumina dental implants have shown good resistance to wear and biocompatibility, but lower strength to flexural forces compared to zirconia [94].

PEEK has been identified as an alternative dental implant material with antibacterial efficacy to Ti or Ti alloys [88]. However, the manufacture of 3D printed PEEK PSIs is limited because PEEK implants are commonly fabricated by processing PEEK rods in bulk, with a higher rate of material waste [88].

Finally, tantalum has been considered a potential dental implant material for its low toxicity, high resistance to corrosion, bone induction, and conduction ability [87]. Wang et al. [87] reported that, in animal models, 3D printed Ta implants fabricated with SLM were better fits compared to conventional Ti implants.

### 7.2. Three-Dimensionally Printed Manufacturing-Controlled Characteristics

The high performance required and the complex shapes of dental implants could be controlled with the advent of additive manufacturing [95]. Customized 3D printed dental implants should be designed tailored to both the implant macrostructure and microstructure, determining and conditioning several implant characteristics and proprieties such as surface roughness, corrosion resistance, and mechanical strength [84,95,96].

Novel dental implant geometries have been 3D printed due to the ability of additive manufacturing to fabricate new and desired implant macrostructures without expensive molds or tools [85,95].

Also, novel 3D printed bioactive dental implants have been produced to improve osteogenesis in bone defects and osteointegration in the implant’s pores, creating regular and size-defined pores in the implant microstructure [84], and an inherent roughness surface through the solidification of melted powder droplets on the implant surfaces.

However, some challenges in 3D printed PSIs have been reported, particularly concerning the manufacturing of PEEK-based PSIs [88]. In fact, the 3D printing selective laser sintering technique produces high PEEK wastage [97]. Moreover, the fused filament fabrication technique has difficulty in printing small PEEK objects (<15 mm), particularly with the threads that characterize dental implants, and in extruding PEEK through a small diameter nozzle for high PEEK viscosity [88]. Finally, at the high temperatures of PEEK printing, the materials become prone to warping and incomplete crystallization, which compromises the dental implant strength and crystallinity [88].

### 7.3. Histological Findings of the Healing Process

Four studies [84,85,86,87] included in the present narrative review evaluated the histological findings after 3D printed dental implants were placed in animal models, considering the range of two weeks [87] to one year [84].

After two weeks of 3D printed dental implant placement, bone growth along the implant pores was found [87]. At four weeks, bone growth was also observed in the center of implant pores [87]. At 3 months, the bone was still actively remodeled around dental implants, but more mature bone morphology was detected.

At one year, trabecular thickness and bone density were high [84]. The trabecular thickness and porosity increased over time, reflecting the physiological bone remodeling process [84].

Although the bone healing process around 3D printed implants is similar to that of traditional dental implants, it must be considered that all of the present data are based on small samples in animal studies; moreover, none of the implants considered had been prosthetically loaded. Dental implant loading (early or delayed) increases bone formation and is a factor that positively influenced osseointegration in traditional dental implants [98].

This represents a further gap in current knowledge about 3D printed dental implants, the validity of which should be further confirmed by studies with humans and with short- and long-term follow-ups.

### 7.4. Complications and Their Management

Tribocorrosion, also known as bio-tribocorrosion in the medical field, is the irreversible degradation process of materials caused by the combined action of mechanical wear (tribo) and the chemically aggressive environment (corrosion), which may lead to metal ion and particle release in the surrounding tissues.

The tribocorrosion process is influenced by many variables, such as the type and the chemical composition of the material, the contact geometry, and external factors, such as environmental temperature and pH [95].

The bio-tribocorrosion process can lead to titanium (Ti) and, to a lesser extent, vanadium (V) and aluminum (Al) ions and particles released from dental implants and implant-supported restorations [99]. Consequently, these products may trigger inflammatory, reactive, and hypersensitive oral manifestations, potentially related to metal particles released from dental implants, including peri-implant mucositis and peri-implantitis [95,99,100].

Selective laser melting (SLM), an additive manufacturing technique for 3D printing, is increasingly utilized for producing dental implants of titanium alloy (Ti6Al4V), due to its ability to create alloys with mechanical properties that are superior or comparable to those of conventional techniques [95,101]. Toptan et al. [102] compared the tribocorrosion of SLM-produced Ti6Al4V alloy with the alloy’s counterparts produced through commercial methods. No statistical differences regarding tribocorrosion process were found in terms of alloys’ total volume loss or volume loss influenced by mechanical wear and wear-accelerated corrosion [95,102].

SLM techniques used to manufacture pure titanium materials enhanced wear resistance in comparison to commercially pure titanium [103]. These promising findings suggested that the SLM technique is capable of producing pure Ti manufacturers with complex shapes, finer grain sizes, and superior wear properties when compared to the casting technique [95,104].

Similar to titanium samples, a CoCrMoW alloy, worked via Laser Metal Fusion, was also compared with wrought LC CoCrMo in Mace and Gilbert’s study. Very close anti-wear responses between them were found, confirming additive manufacturing medical tools as a valid alternative again [95,105]. Zhang et al. [58] stated that a 3D zirconia sample not only reached a satisfactory level of mechanical resistance, comparable to the tools realized by subtractive manufacturing methods, but also enhanced cellular activity. In fact, the peculiar surface patterns with directional pores promoted the osteoblast response, whereas the dense core had long-term mechanical resistance [58,95].

Despite the great potential of this technology, some drawbacks, unfortunately, exist, such as the impossibility of creating nanoscale or bioactive surfaces without a subsequent treatment [106] as well as the absence of standard protocols [107] and of a biological and medical long-term response [95,108].

### 7.5. Clinical Considerations

The main advantage of 3D printed PSIs versus traditional dental implants is the specific fit to the bone defect size [93].

Three-dimensionally printed PSIs could improve primary stability due to its geometry and and can be loaded earlier, reducing the treatment time and the number of patient visits [88,93].

Other studies on beagle dogs have evaluated the different outcomes after implant surgery using three different implant designs: traditional threaded implants, 3D printed implants without spikes, and 3D printed implants with spikes [86]. The 3D printed implants were characterized by the presence of a lattice structure and were designed with or without spikes to compare the spikes’ influences on the 3D printed implant’s stability [86]. The lattice structures of the 3D printed dental implants were used to increase the implant surface area and to promote the new bone growth into the implant pores [86]. The primary stability was lower in the group of 3D printed implants with spikes than in the group of threaded implants. Moreover, at 12 weeks after surgery, no significant difference in stability was found, and the bone-to-implant contact and the bone area fraction occupied were comparable between the three groups [86]. These results showed that the lattice structure in the 3D printed implants did not influence the stability, which was similar to the traditional threaded implants [86]. The authors suggested that the lower implant stability of 3D printed implants with spikes was linked to the larger osteotomy necessary for the spikes during the surgery, which created a gap between the implant surfaces and the surgical bed [86].

At the current stage of knowledge, the mechanical and biological properties of PSIs are promising, but further studies are needed to evaluate a wide range of properties and materials, varying the chemical and tribological environment such as te kind of solution, motion regime, and contact pressure and forces [95].

The main limitation concerning PSIs is the absence of human studies, which precludes the possibility to evaluate patient-reported outcomes. Human studies are necessary to assess the patient-reported outcomes of 3D printed dental implants to assess their behavior in vivo and not only with regard to mechanical characteristics.

Furthermore, human studies should investigate clinical long-term follow-ups, so current knowledge is mainly limited to in vitro and animal studies [95].

## 8. Challenges and Future Prospectives

The present narrative review aimed to elucidate the digital radiographic process in the 3D printing workflow, materials, clinical indications, 3D printed manufacturing-controlled characteristics, histological findings of healing processes, possible complications and their management, patient-reported outcomes, and clinical considerations at short- and long-term follow-up of available customized mesh, membranes, bone substitutes, and 3D printed dental implants applied to GBR in oral implantology.

The 3D-printed technology applied to GBR in oral implantology was associated with encouraging clinical outcomes concerning meshes, membranes, and bone grafts, while no clinical data on humans were available for dental implants.

Customized 3D printed meshes showed several advantages, such as quicker surgery, personalized fitting, reduction in the pain to obtain mesh retention, smoother edges, less mucosal stress, and the possibility of filling the 3D printed bone graft outside the oral cavity, reducing the risk of graft intraoral dissemination or contamination [2,26].

Customized 3D printed titanium meshes were the most utilized in GBR surgery due to the registered bone augmentation rate in width and height of the alveolar ridge associated with an exposure rate similar to that of traditional mesh [26].

The 3D printed zirconia mesh has shown more concerns than titanium mesh regarding material fragility and the related fracture risk [13].

Customized 3D printed membranes fabricated with biocompatible polymers have shown superior or comparable properties to collagen membranes and the possibility to link the biodegradability, tissue integration ability, and biocompatibility of the resorbable membranes, with superior mechanical stability, longevity, and space maintenance properties of the non-resorbable membranes [60]. The ratio of the polymers constituting the 3D printed membranes plays an important role in determining the mechanical properties and the rate of biodegradation, and thus their barrier properties during the healing process over time [56].

Customized 3D printed PLGA (70:30) and pure PCL membranes with or without β-TCP were suitable for GBR surgery, in contrast to PLGA (10:90) membranes or pure PGA membranes for the high resorption rate that led to the collapse of the membrane in the defect [52,55]. The addition of β-TCP with synthetic polymers allows for the enhancement of low polymer bioactivity by releasing ions calcium, which induces bone regeneration and increases the membrane elastic modulus and surface roughness [52].

Customized 3D printed bone substitutes have shown good suitability for bone defects, shortened operation time, unnecessary autologous bone graft harvest, reduction in bone material waste, and the possibility to imitate both the cortex and trabecular structure of the alveolar bone [70,71]. Three-dimensionally printed nano-porous hydroxyapatite bone substitutes, with or without β-TCP [69,75], showed no difference from commercial bone substitutes in bone regeneration [70].

Customized 3D printed dental implants have shown high capability in matching the anatomy of the bone defect, providing primary stability, facilitating osseointegration, and reducing the amount of peri-implant bone graft. However, current knowledge is limited to in vitro or animal studies, and no studies have compared the different materials [85,88,89,91,92].

The development of new materials for dental implants with specific coatings should be explored, and further clinical human studies are needed. Further studies concerning customized 3D printed meshes and membranes should also investigate the short- and long-term follow-up of peri-implant bone levels and volumes after prosthetic functional loading.

Three-dimensional technology has shown several advantages when applied in GBR surgery; however, it is associated with an initial increased economic cost. In the future, the continued development of software and 3D manufacturing machines could support a reduction in the initial costs of 3D technology [109].

In addition, conversely to simple 3D printed customized products, more complex designs have higher costs and require more fabrication time [109].

Technological development should be accompanied by advances in the study of new biocompatible 3D printed materials, particularly in improving the mechanical properties of 3D printed dental implants, which also requires further long-term clinical data [9].

Furthermore, while 3D printing technology is developing, other progressions in 3D-printable smart materials have led to a newer generation of four-dimensional (4D) printing, in which the fourth dimension is time combined with 3D printing.

Four-dimensionally printed products can change their shape and/or mechanical characteristics after being printed in response to several surrounding media [94].

The use of 4D printing technology in implantology has been proposed for the replacement of titanium alloys, for which hypersensitivity reactions have been reported, with biocompatible shape memory polymers in order to also achieve a better elastic deformation capacity of the implant, cost reduction, lower density, and thus lower weight, ease of production, programming customization, and biocompatibility [94].

## 9. Conclusions

The new frontiers of computer-based surgery and its applications in customized 3D printed GBR in oral implantology were investigated to customize implant-prosthetic rehabilitations according to patient-specific characteristics and needs, achieving high rates of functional and esthetic success and reducing the intra-operative duration of and the risk of complications, starting from accurate pre-operative planning favored by the use of the technology itself.

Therefore, the present narrative review aimed to elucidate the digital radiographic process in the 3D printing workflow, materials, clinical indications, 3D printed manufacturing-controlled characteristics, histological findings of healing processes, possible complications and their management, patient-reported outcomes, and clinical considerations at short- and long-term follow-up of available customized mesh, membranes, bone substitutes, and 3D printed dental implants applied to GBR in oral implantology.

Customized 3D printed titanium meshes were considered a successful solution in GBR surgery for the augmentation of horizontal and vertical bone, as well as for regenerating complex bone defects in the esthetic area. Relevant bone augmentation in width (up to 11.48 mm, with a mean of 6.35 mm) and in height (up to 8.90 mm, with a mean of 4.78 mm) were recorded using 3D printed Ti meshes.

Three-dimensionally printed synthetic polymer membranes had the main characteristic of linking the advantages of conventional resorbable and non-resorbable membranes. Most studies showed superior or comparable mechanical and biological properties of 3D printed membranes compared to collagen membranes. However, most of the currently available data on 3D printed membranes have resulted from animal studies.

Customized 3D printed bone substitutes’ main advantage is the possibility of manufacturing a customized surface and porous structures, unnecessary autologous bone graft harvest, and manual modification, resulting in the minimization of contamination risk and the reduction in bone material waste. Moreover, the bone substitute architectural design can imitate both the cortex and trabecular structure of the alveolar bone.

Few data on customized 3D printed dental implants and abutments are available, but in vitro and animal studies have reported promising results for new designs that could improve their mechanical properties and tribocorrosion-associated complications.

Current evidence shows the potential of 3D printing technology in GBR; in particular, based on current knowledge, the most promising results seem to be for 3D printed meshes. However, further human studies investigating the short- and long-term follow-up of peri-implant bone levels and volumes after prosthetic functional loading are necessary.

## Figures and Tables

**Table 1:** Data extracted and collected concerning customized 3D printed mesh applied to GBR in oral implantology surgery. Studies: First Author, reference, years and journal of publication, study’s design, sample size. Three-dimensionally printed mesh: material and number of manufactured mesh, 3D printing method, type of bone defect, position (maxilla/mandible), associated membrane and bone substitute. Three-dimensionally printed manufacturing-controlled characteristics (e.g., shape, pore size, roughness). Clinical and radiographical findings at any time point. Histological findings of the healing process at any time point. Reported complications at any time point. Patient-reported outcomes (e.g., pain, esthetic satisfaction). The main study’s conclusions.

Studies	Three-Dimensionally Printed Mesh	Three-Dimensionally Printed Manufacturing-Controlled Characteristics	Clinical and Radiographical Findings	Histological Findings of the Healing Process	Reported Complications	Patient-Reported Outcomes	Main Conclusions
Human Studies							
Boogaard M., [25] 2022 Comped Contin Educ Dent Human study (case series) Sample size: n.2	Material: Ti (n.2) Three-dimensional printing method: MD Bone defect: horizontal and vertical (n.2) Position: mandibular posterior region (n.2) Associated membrane: pericardium membrane (CopioOs^®^) (n.2) Associated bone substitute: particulate allograft (Puros^®^) (n.1); autologous bone and xenograft (RegenerOss^®^) (n.1)	Shape	After 6 months Mean vertical bone gain: 5.4 mm (range 4.1–6.7 mm) Mean horizontal bone gain: 9.75 mm (range 8.7–10.8 mm)	MD	Mesh exposure (n.1)	MD	The GBR surgery utilized customized 3D printed Ti meshes that were safe and predictable in horizontal and vertical ridge augmentations and could be associated with direct implant placement.
Chiapasco M., [26] 2021 Clin Oral Implants Res Human study (retrospective study) Sample size: n.41	Material: Ti (n.53) Three-dimensional printing method: laser sintering (n.53) Bone defect: MD Position: N/D Associated membrane: collagen membrane (n.53) Associated bone substitute: 50:50 autologous bone and xenograft (Bio-Oss^®^) (n.53)	Shape	After 7 months Mean vertical bone gain: 4.78 ± 1.88 mm (range 1.00–8.90 mm) Mean horizontal bone gain: 6.35 ± 2.10 mm (range 2.14–11.48 mm) After 10 months Implant survival rate: 100%	No signs of necrosis, fatty cell infiltration, or fibrosis were observed	Early mesh exposure (n.11) Mesh infection (n.1) Partial bone loss (n.4) Need to remove mesh earlier (n.2)	MD	The 3D printed Ti mesh seemed to represent a reliable GBR approach for severe atrophic edentulous ridge defects, in terms of mean vertical bone gain, peri-implant bone resorption, and survival implant rates. The 3D printed Ti mesh simplified the surgery but was associated with a higher mesh exposure and complexity in mesh removal.
Ciocca L., [27] 2018 J Oral Implantol Human study (prospective study) Sample size: n.9	Material: Ti (n.9) Three-dimensional printing method: direct metal laser sintering (n.9) Bone defect: MD Position: MD Associated membrane: none Associated bone substitute: 50:50 autologous bone from the iliac crest or mandibular ramus and xenograft (Bio-Oss^®^) (n.9)	Shape Thickness	After 6–8 months Vertical bone gain: range 1.72–4.1 mm in mandible; 2.14–6.88 in maxilla	MD	Early mesh exposure (n.3) Late mesh exposure (n.3) Mesh infection (n.1)	MD	The 3D printed Ti mesh should be considered a cautious approach due to the high post-operative morbidity rate for mesh exposure. The number of retaining screws was reduced to one or at most two.
Cucchi A., [28] 2021 Clin Oral Implants Res Human study (RCT) Sample size: n.30	Material: Ti (n.30) Three-dimensional printing method: SLM (n.30) Bone defect: vertical (n.25) horizontal (n.5) Position: maxillary anterior region (n.6); mandibular anterior region (n.1); maxillary posterior region (n.8) mandibular posterior region (n.15) Associated membrane: none (n.15); collagen membrane (n.15) Associated bone substitute: 50:50 autologous bone and xenograft (Zcore^®^) (n.30)	Shape Roughness	After 6 months Bone density: hard (n.7); medium (n.19); soft (n.4) Pseudo-periosteum: type 1 (n.17); type 2 (n.8); type 3 (n.5) Mean regenerated bone volume: 40.07 mm^3^ Mean lacking bone volume: 37.4 mm^3^ Mean regeneration rate: 7.98%	MD	Paresthesia of mental nerve (n.3) Paresthesia of infra-orbital nerve (n.1) Partial mesh fractures (n.2) Partial mesh misfitting (n.1) Early mesh exposure (n.3) Late mesh exposure (n.2) Mesh infection (n.2)	MD	The 3D printed Ti mesh can be considered a valid solution to horizontal and vertical bone regeneration, both in using mesh alone or covered with a long-lasting membrane. The use of 3D printed Ti mesh alone was comparable to using the mesh covered by a membrane. No statistically significant differences were found, even if a better trend in regeneration rates and healing complications was found when the Ti mesh was combined with a membrane.
Dellavia C., [29] 2021 Clin Implant Dent Relat Res Human study (cohort study) Sample size: n.20	Material: Ti (n.20) Three-dimensional printing method: laser sintering (n.20) Bone defect: vertical and horizontal (n.20) Position: MD Associated membrane: collagen membrane (n.20) Associated bone substitute: 50:50 autologous bone and xenograft (Bio-Oss^®^) (n.20)	Shape	After 9 months Mean vertical bone gain: 5.20 mm Mean horizontal bone gain: 6.80 mm Mean vertical bone resorption: 0.35 mm Mean horizontal bone resorption: 0.34 mm	After 9 months Regenerated bone showed high organization and mineralization and close contact with Ti mesh and was characterized by medullary spaces, blast-like and osteoid cells in the periphery region, rare inflammatory cells, and blood vessels	Early mesh exposure (n.2) Late mesh exposure (n.1) Mesh infection (n.0)	MD	Customized 3D printed Ti meshes can be applied in GBR surgery with histological findings that make the regenerated bone suitable for implant placement. Three-dimensionally printed mesh exposures did not significantly affect the regenerated bone.
De Santis D., [30] 2022 J Clin Med Human study (case series) Sample size: n.10	Material: Ti (n.10) Three-dimensional printing method: laser sintering (n.10) Bone defect: less than 8 mm in height and 5 mm in thickness Position: from maxillary incisor to first premolar (n.6); mandibular molars (n.4) Associated membrane: collagen membranes (n.10) Associated bone substitute: 50:50 autologous bone and xenograft (Bio-Oss^®^) (n.20)	Shape	At 6 months Mean horizontal bone gain: 6.37 ± 2.17 mm (range 2.78–9.12 mm) Mean vertical bone gain: 5.95 ± 2.06 mm (range 2.68–9.02 mm) Mean regenerated bone volume: 3012 ± 1938 mm^3^ (range 1273–6879 mm^3^)	MD	Late mesh exposure (n.1)	MD	Customized bone regeneration with customized titanium meshes is a predictable and encouraging alternative to traditional GBR.
Geletu G.L., [31] 2022 Medicina (Kaunas) Human study (case report) Sample size: n.1	Material: Ti (n.1) Three-dimensional printing method: SLM (n.1) Bone defect: MD Position: maxillary lateral incisor and canine Associated membrane: none Associated bone substitute: 50:50 mixed particulate bone of allograft bone and pure mineral bovine	Shape	After 11 months Vertical bone gain: 11.63 mm Horizontal bone gain: 10.34	MD	None	MD	The customized 3D printed Ti mesh is easy to manipulate, reduces surgery time, has a low risk of dehiscence, and is predictable in volume bone regeneration.
Inoue K., [24] 2018 Implant Dent Human study (case series) Sample size: n.2	Material: Ti (n.2) Three-dimensional printing method: SLM (n.2) Bone defect: MD Position: maxillary central incisor (n.1); from maxillary canines to second premolar (n.1) Associated membrane: none (n.1); collagen membrane (n.1) Associated bone substitute: xenograft (Bio-Oss^®^) (n.1); autologous bone (n.1)	Shape Thickness Porous size	MD	MD	None	MD	The 3D printed Ti meshes manufactured through SLM 3D printed methods can be applied to several bone defect types, reducing the surgery time and post-operative infection risk.
Lizio G., [32] 2022 Clin Oral Implants Res Human study (pilot study) Sample size: n.17	Material: Ti (n.17) Three-dimensional printing method: laser sintering (n.17) Bone defect: vertical and horizontal (n.17) Position: maxillary anterior region (n.7); maxillary posterior region with sinus lift (n.1); mandibular posterior region (n.11) Associated membrane: none Associated bone substitute: 60:40 autologous bone and xenograft (Bio-Oss^®^) (n.17)	Shape Thickness	After 6 months Mean regenerated bone volume: 1003.92 mm^3^ Mean lacking bone volume: 149.33 mm^3^ Mean regeneration rate: 83.99%	MD	Paresthesia of mental nerve (n.1) Partial mesh misfitting (18%) Early mesh exposure (n.6) Late mesh exposure (n.4) Mesh infection (n.3) Loss of graft: (n.5)	MD	The 3D printed Ti meshes improved the predictability of GBR by up to 88% in 74% of cases.
Mounir M., [33] 2019 Clin Implant Den Relat Res Human study (RCT) Sample size: n.8	Material: PEEK (n.8) Three-dimensional printing method: MD Bone defect: vertical and horizontal (n.8) Position: N/D Associated membrane: collagen membrane (n.8) Associated bone substitute: 50:50 autologous bone from the anterior iliac crest and xenograft (n.8)	Shape Thickness	After 6 months Mean regeneration rate: 31.8%	MD	Early mesh exposure (n.1)	MD	The customized 3D printed PEEK mesh could be used as a valid product in GBR surgery. No statistically significant differences were found compared to a prebent Ti mesh
Navarro Cuéllar C., [34] 2021 J Clin Med Human study (retrospective study) Sample size: n.8	Material: Ti (n.8) Three-dimensional printing method: MD Bone defect: horizontal (mean 10.05 cm) and vertical Position: MD Associated membrane: none Associated bone substitute: cortico-cancellous iliac crest graft and free fibula flap (n.8)	Shape Pore size Roughness	After 6 months Mean vertical bone gain: 12.22 mm (range 10.1–13.4 mm) Implant survival rate (94.7%) After 3 years of prosthetic rehabilitation Mean bone resorption: 1.43 mm (range 0.5–2.4 mm)	MD	None	Good esthetic result (n.6) Fair results (n.2)	The combination of virtual surgical planning and 3D printed Ti meshes increased the intra-operative efficiency and reduced the complication rate and bone resorption.
Nickenig H.J., [35] 2022 Clin Oral Investig Human study (case series) Sample size: n.3	Material: Ti (n.3) Three-dimensional printing method: MD Bone defect: buccal bone concavities (mean 4.00 mm) Position: anterior region of the maxilla (n.1) or mandibula (n.2) Associated membrane: none Associated bone substitute: 2/3 or 1/3 ratio of autologous bone and xenograft (Bio-Oss^®^)	Shape	After 6 months Mean bone gain: 3.7 ± 0.59 mm (range 3.1–4.8) After 12 months Mean bone gain: 4.3 ± 0.83 (range 3.2–5.1 mm)	MD	None	MD	Customized 3D printed Ti meshes are highly reliable in terms of augmentation extent and healing when used for the GBR of buccal bone concavities of the anterior alveolar ridge.
Tallarico M., [1] 2020 Materials (Basel) Human study (case report) Sample size: n.1	Material: Ti (n.1) Three-dimensional printing method: MD Bone defect: horizontal Position: maxillary right central incisor Associated membrane: none Associated bone substitute: 50:50 autologous bone and xenograft (Bio-Oss^®^)	Shape	After 12 months Implant survival rate: 100%	MD	None	MD	A 3D printed Ti mesh can be manufactured with higher accuracy and could represent a valid option for GBR surgery associated with predictable results.
Yang, W., [36] 2022 BMC Oral Health Human study (retrospective study) Sample size: n.20	Material: Ti (n.20) Three-dimensional printing method: laser additive manufacturing (n.20) Bone defect: minor bone defect less or equal to 150 mm^2^ (n.10); major bone defects greater than 150 mm^2^ (n.10); vertical (n.6); horizontal (n.2); vertical and horizontal (n.12) Position: maxilla (n.7); mandible (n.13) Associated membrane: i-PRF and N/D resorbable membrane (n.20) Associated bone substitute: 50:50 autologous bone and xenograft	Shape Thickness Inner diameter of titanium pin holes	MD	MD	Late mesh exposure (n.3) Mesh infection (n.0)	MD	The 3D printed Ti mesh accuracy was not significantly affected by alveolar bone defect size.

Abbreviations: three-dimensional, “3D”; number, “n”; randomized clinical trial, “RCT”; Titanium, “Ti”; poly-ether-ether-ketone, “PEEK”; selective laser melting, “SLM”; guided bone regeneration, “GBR”; platelet-rich fibrin, “PRF”; injectable platelet-rich fibrin, “i-PRF”; missing data, “MD”; not defined, “N/D”.

**Table 2:** Data extracted and collected concerning customized 3D printed membranes applied to GBR in oral implantology surgery. Studies: First Author, reference, years and journal of publication, study’s design, sample size. Three-dimensionally printed membrane: material and number of manufactured membranes, 3D printing method, type of bone defect, position (maxilla/mandible), associated mesh and bone substitute. Three-dimensionally printed manufacturing-controlled characteristics (e.g., shape, pore size, roughness). Clinical and radiographical findings at any time point. Histological findings of the healing process at any time point. Reported complications at any time point. Patient-reported outcomes (e.g., pain, esthetic satisfaction). The main study’s conclusions.

Studies	Three-Dimensionally Printed Membrane	Three-Dimensionally Printed Manufacturing-Controlled Characteristics	Clinical and Radiographical Findings	Histological Findings of the Healing Process	Reported Complications	Patient-Reported Outcomes	Main Conclusions
Human study and ex vivo study							
Manzano Romero P., [57] 2021 J Craniofac Surg Human study and ex vivo (retrospective) Sample size: n.13	Material: PLA n.12 Three-dimensional printing method: fused deposition modeling (n.13) Bone defect: horizontal (n.10); vertical (n.3) Position: maxilla (n.10); mandible (n.3) Associated mesh: none Associated bone substitute: xenograft (n.10)	Shape	Mean horizontal bone gain: 8.29 mm Mean vertical bone gain: 6.96	MD	MD	NA	A 3D printed model is a valid tool for planning GBR surgery. Customized surgery can improve the students’ and surgeons’ training, as well as the communication with patients, and help dentists visualize during the operation.
Animal Studies							
Jamalpour M.R., [50] 2022 Dent Mater Animal study (canine) Sample size: n.4	Material: PCL and hydrogen (n.6) Three-dimensional printing method: MD Bone defect: 5.8 mm in diameter, 2 mm in depth (n.8) Position: tibia (n.4) Associated mesh: Associated bone substitute: xenograft (Cerabone^®^) (n.8)	Shape Pore size Biodegradability	After 4 weeks Mean regenerated bone gain (large pore size membrane): 60.00% Mean regenerated bone gain (small pore size membrane): 55.71%	After 4 weeks Mean fibrous tissue (large pore size membrane): 40.00% Mean fibrous tissue (small pore-size membrane): 44.29%	MD	NA	A 3D printed hybrid bi-layered membrane in Gelatin/PCL could represent a promising membrane for GBR surgery due to its interfacial tissue good properties.
Jang H.J., [51] 2023 Nanoscale Adv Animal study (rat) Sample size: n.24	Material: graphene oxide-incorporated PLA (n.24) Three-dimensional printing method: MD Bone defect: 5 mm in diameter Position: calvaria Associated mesh: MD Associated bone substitute: MD	PLA concentration Mechanical properties Biodegradability	After 4 weeks Bone mineral density: 1.30 ± 0.07 g/cm^3^ After 8 weeks Bone mineral density: 1.19 ± 0.04 g/cm^3^	MD	MD	NA	The graphene oxide-incorporated PLA membranes exhibited excellent physico-chemical properties and provide a microenvironments= that can facilitate the preosteoblasts’ behaviors.
Kim E.V., [53] 2021 Bull Expo Biol Med Animal study (rabbit) Sample ize: n.36	Material: PLA (n.18) and PLA/PGA (n.18) Three-dimensional printing method: layer-by-layer deposition Bone defect: MD Position: right ear Associated mesh: none Associated bone substitute: cortico-cancellous iliac crest graft and free fibula flap (n.8)	Shape Thickness Roughness	MD	After 7 days A thin connective capsule was detected in the dermis around PLA membranes, with lots of macrophages, moderate and diffuse infiltration of plasmacells, and no vascular cells. Fibrous connective tissue was detected in the dermis around PLA/PGA membranes separated by layers of collagen, granulation tissue, and lymphoplasmacytic infiltration in small foci and few macrophages. Copolymer swelling and impregnation. No vascular ingrowth. After 14 days Foreign-body giant cells were detected on the PLA membrane surface. Intensification of PLA/PGA swelling and impregnation was observed. After 28 days A connective tissue capsule was detected around the PLA membrane, and a lot of macrophages and lymphoplasma cells were inside and under the capsule. No vascular ingrowth. A fibrous connective tissue, separated by a dense fibrous connective tissue, was found around the PLA/PGA membrane.	MD	NA	The 3D printed PLA/PGA membranes showed more promising results than 3D printed PLA membranes for their periods of bioresorption, and a gentler hydrolytic decomposition process for the surrounding tissues.
Lee J.Y., [55] 2021 Materials (Basel) Animal study (rabbit) Sample size: n.10	Material: PCL (n.10); PCL/β-TCP (n.10) Three-dimensional printing method: melting process (n.20) Bone defect: n.20 Position: calvaria (n.30) Associated mesh: none Associated bone substitute: 60:40 hydroxyapatite and β-TCP (n.10)	Shape Thickness	After 2 weeks Mean regenerated bone volume (PCL): 3.34 ± 1.88 mm^3^ Mean regenerated bone volume (PCL/β-TCP): 28.94 ± 8.15 mm^3^ After 8 weeks Mean regenerated bone volume (PCL): 20.41 ± 5.75 mm^3^ Mean regenerated bone volume (PCL/β-TCP): 62.20 ± 21.58 mm^3^	After 2 weeks In the PCL/β-TCP group, new bone was found at the defect margins. No inflammatory cells were detected. In the PCL group, new bone was found from the defect margin and around the graft particles. After 8 weeks Some parts of the PCL/β-TCP membranes were resorbed and replaced by new bone and connective tissue. No inflammatory cells were detected. More mature lamellar bones were observed compared to 2 weeks. In the PCL group, new bone was found across the defect. No inflammatory cells were detected. The membrane was intact and integrated with the surrounding tissue.	None	NA	Three-dimensionally printed PCL/β-TCP membranes showed good structural stability, slow degradation, and biocompatibility. The greatest regenerated bone volume was obtained when the membrane was associated with the bone substitute.
Petposri S., [56] 2023 J Funct Biomater Animal study (rat) Sample size: MD	Material: PLGA (LA:GA = 10:90) (n.MD); PLGA (LA:GA = 70:30) (n.MD) Three-dimensional printing method: SLM (n.MD) Bone defect: 8 mm in diameter Position: calvaria Associated mesh: MD Associated bone substitute: MD	Shape PLA and PGA concentration Microstructures Mechanical properties Biodegradability Pore sizes Thickness	After 2–4 weeks The new bone in PLGA (70:30) was lower than that of the group with a collagen membrane. After 8 weeks The new bone in PLGA (70:30) was greater than that of the group with a collagen membrane.	After 2 weeks Dense fibrous tissue was found surrounding PLGA (70:30) membrane and new bone in the middle and from the peripheries of the bone defect. After 4 weeks New bone regenerating from the defect peripheries; occasionally, giant cells with ingesting PLGA (70:30) membrane particles were found. After 8 weeks New bone regenerating along the inferior side of the PLGA (70:30) membrane.	Membrane infection (n.0) Dehiscence (n.0) Lack of mechanical properties over time (PLGA 10:90) Acid products (PLGA 10:90)	NA	Three-dimensionally printed PLGA (70:30) membranes were suitable for GBR surgery due to their good degradability, biocompatibility, and mechanical properties. The viability of cells cultured on 3D printed PLGA (10:90) membranes decreased after one weeks. For this reason, PLGA (10:90) was not suitable for GBR surgery.
Shim J.H., [59] 2017 Int J Mol Sci Animal study (beagle) Sample size: n.3	Material: PCL (n.3) or PCL/β-TCP (n.3) Three-dimensional printing method: multi-head deposition system Bone defect: length, 7 mm; height, 5 mm; depth, 5 mm (n.18) Position: mandible (n.3) Associated mesh: none Associated bone substitute: deproteinized bovine bone grafts (n.3)	Shape Thickness Pore size Mechanical properties	After 8 weeks Mean regenerated bone volume (PCL): 27.29 ± 2.19 mm^3^ Mean regenerated bone volume (PCL/β-TCP): 29.22 ± 3.11 mm^3^ Mean non-mineralized tissue volume (PCL): 123.58 ± 5.56 mm^3^ Mean non-mineralized tissue volume: (PCL/β-TCP): 122.37 ± 7.33 mm^3^ Mean remaining bone substitute volume: (PCL): 24.84 ± 5.30 mm^3^ Mean remaining bone substitute volume: (PCL/β-TCP): 24.12 ± 5.48 mm^3^	After 8 weeks PCL and PCL/β-TCP membranes were closely in contact with the buccal bone. New bone was observed around the xenograft graft.	None	NA	The 3D printed PCL membranes, as well as the PCL/β-TCP membranes, showed better GBR performance compared to collagen membrane. 3D printed PCL/β-TCP showed greater structural stability, and their potential use as a resorbable membrane in GRB surgery should be considered.
Won J.Y., [52] 2016 Biomed Mater Animal study (beagle) Sample size: n.3	Material: PCL/PLGA/ β-TCP (n.MD) Three-dimensional printing method: multi-head deposition system (n.MD) Bone defect: n.6 Position: first and second premolars region Associated mesh: none Associated bone substitute: deproteinized bovine bone grafts	Shape Roughness Mechanical properties	After 8 weeks Mean regenerated bone volume: 1.57 ± 0.70 mm^3^ Mean non-mineralized tissue volume: 8.52 ± 2.47 mm^3^ Mean remaining bone substitute volume: 3.95 ± 1.97 mm^3^ Bone-to-implant contact: 56.48 ± 4.68%	After 8 weeks The newly bone was formed in the buccal implant area. A large amount of material grafts was found in the peri-implant dehiscence area.	None	NA	Three-dimensionally printed PCL/PLGA/ β-TCP membranes showed similar performance in bone regeneration compared to collagen membranes applied to GBR surgery in peri-implant defects. Hence, 3D printed PCL/PLGA/ β-TCP membranes had good efficacy as resorbable GBR membranes and with a higher stability than collagen membranes.
In vitro study							
Zhang H.Y., [58] 2019 Materials (basel) In vitro study Sample size: n.MD	Material: PLA (n.MD) Three-dimensional printing method: fused deposition modeling (n.MD)	Thickness Pore size	NA	NA	None	NA	Three-dimensionally printed membranes showed better mechanical properties than conventional membranes manufactured via the conventional solvent casting process. The membrane pore size (small, large, or no pore) conditioned the mechanical properties and cell growth.

Abbreviations: three-dimensional, “3D”; number, “n”; poly lactic acid, “PLA”; poly lactic-co-glycolic acid, “PLGA”; polycaprolactone, “PCL”; beta-tricalcium phosphate, “β-TCP”; selective laser melting, “SLM”; guided bone regeneration, “GBR”; missing data, “MD”; not defined, “N/D”; not applicable, “NA”.

**Table 3:** Data extracted and collected concerning customized 3D printed bone substitutes applied to GBR in oral implantology surgery. Studies: First Author, reference, years and journal of publication, study’s design, sample size. Three-dimensionally printed bone substitutes: material and number of manufactured bone substitutes, 3D printing method, type of bone defect, position (maxilla/mandible), associated mesh and membranes. Three-dimensionally printed manufacturing-controlled characteristics (e.g., shape, pore size, roughness). Clinical and radiographical findings at any time point. Histological findings of the healing process at any time point. Reported complications at any time point. Patient-reported outcomes (e.g., pain, esthetic satisfaction). The main study’s conclusions.

Studies	Three-Dimensionally Printed Bone Substitutes	Three-Dimensionally Printed Manufacturing-Controlled Characteristics	Clinical and Radiographical Findings	Histological Findings of the Healing Process	Reported Complications	Patient-Reported Outcomes	Main Conclusions
Human study							
Kijartorn P., [68] 2017 Key Engineering Materials Human study (case series) Sample size: n.5	Material: HA (n.5) Three-dimensional printing method: MD Bone defect: MD Position: maxillary anterior region (n.5) Associated mesh: none Associated membranes: collagen membrane (Bio-gide^®^)	Granule size	At 8 weeks Good primary stability (n.5)	At 8 weeks Few neutrophils were found. The grafted area was occupied by newly regenerated bone encompassing or in close contact with residual HA granules and tissue of granulation. Blood vessels, osteoblast, and marrow tissue were observed.	None	MD	Three-dimensionally printed HA bone substitutes may represent a suitable alternative.
Kijartorn P., [69] 2022 J Dent Sci Human study (RCT) Sample size: n.30	Material: HA (n.15) Three-dimensional printing method: MD Bone defect: MD Position: MD Associated mesh: none Associated membranes: non-resorbable membrane (n.15)	Granule size	After 3 months Implant stability: 73.8 ± 2.87	After 4 months Mean bone formation: 33.20 ± 6.73% Mean residual bone graft: 27.04 ± 7.91% Mean connective tissue: 39.76 ± 4.03%		MD	Three-dimensionally printed HA bone substitutes may represent a suitable alternative.
Kim N.H., [70] 2024 Sci Rep Human study (prospective RCT) Sample size: n.60	Material: 60:40 HA and β-TCP (n.30) Three-dimensional printing method: digital light sintering (n.30) Bone defect: MD Position: MD Associated mesh: none Associated membranes: collagen membrane (n.30)	Shape Pore size	After 5 months Mean tissue volume: 44.87 ± 5.59 mm^3^ Mean bone volume: 8.94 ± 2.32 mm^3^ Bone surface density: 14.95 ± 6.39 L/mm^3^ Bone mineral density: 0.77 ± 0.46 g/cm^3^	After 5 months New bone was found around the bone substitutes. No specific inflammatory responses were detected around the bone substitutes.	MD	No discomfort after surgery (n.30)	Customized 3D printed bone substitutes are a potential alternative to conventional bone substitutes in GRB surgery. However, HA and β-TCP did not demonstrate significant regeneration properties compared to conventional bone substitutes.
Mekcha P., [12] 2023 J Prosthodont Res Human study (case series) Sample size: n.12	Material: nanoHA (n.12) Three-dimensional printing method: MD Bone defect: horizontal (n.12) Position: anterior region (n.6); premolars region (n.4); molar region (n.4) Associated mesh: MD Associated membranes: PRF (n.9)	Shape Internal microstructure Pore size	After 6 months Mean horizontal bone gain: 2.45 ± 0.70	After 6 months Newly regenerated bone and blood vessels were retrieved at the graft interface and native bone area. Mean bone tissue: 30.48 ± 4.81% Mean new bone formation: 28.6 0 ± 1.88% Mean residual graft: 19.82 ± 4.07% Mean connective tissue: 20.81 ± 4.41%	Dehiscence (n.2) Partial/total bone graft failure (n.3)	During surgical time VAS score: 1.41 ± 0.51 After 2 weeks VAS score: 0.92 ± 0.51 After 1 month VAS score: 0.33 ± 0.49 After 2, 3, 6 months VAS score: 0	The 3D printed HA bone substitute is a viable option for primary implant-site regeneration.
Animal study							
Kim J.W., [71] 2020 Int J Mol Sci Animal study (beagle) Sample size: n.12	Material: HA and β-TCP (n.16) Three-dimensional printing method: digital light processing Bone defect: 7 mm × 3 mm × 6 mm (n.16) Position: mandibular second premolar and first molar region (n.16) Associated mesh: none Associated membranes: collagen membrane (Genoss^®^) (n.16)	Shape Mechanical properties	After 4 weeks Mean new bone formation: 27.44 ± 5.86% Mean residual graft: 31.02 ± 1.35% After 8 weeks Mean residual graft: 67.72 ± 11.25%	After 4 weeks No signs of inflammation. Granulation tissue was observed in the defects. New blood vessels were found. After 8 weeks No signs of inflammation. Little/no granulation tissue was observed in the defects. New blood vessels were found. New bone ingrowth on the lower and center side.	None	NA	The mechanical properties and bone regenerative ability of 3D printed HA/β-TCP bone substitutes could be affected by pore structure. Three-dimensionally printed HA/β-TCP bone substitutes are easy to use in large defect area and have good bone-forming ability.
In vitro study							
Thammarakcharoen F., [72] 2015 Key Engineering Materials In vitro study Sample size: n.MD	Material: HA (n.MD) Three-dimensional printing method: MD	Resorbability Microstructure Pore size	NA	NA	None	NA	Crystallinity and porosity of 3D printed HA bone substitutes are important characteristics that conditioned the resorbability rate. High porosity combined with low crystallinity were preferred to enhance the resorbability.

Abbreviations: three-dimensional, “3D”; number, “n”; poly-ether-ether-ketone, “PEEK”; beta-tricalcium phosphate, “β-TCP”; hydroxyapatite, “HA”; platelet-rich fibrin, “PRF”; selective laser melting, “SLM”; guided bone regeneration, “GBR”; randomized controlled trial, “RCT”; visual analog scale, “VAS”; missing data, “MD”; not defined, “N/D”; not applicable, “NA”.

**Table 4:** Data extracted and collected concerning customized 3D printed endosseous dental implants applied to GBR in oral implantology surgery. Studies: First Author, reference, years and journal of publication, study’s design, sample size. Three-dimensionally printed dental implant: material and number of manufactured dental implants, 3D printing method, type of bone defect, position (maxilla/mandible), associated membrane and bone substitute. Three-dimensionally printed manufacturing-controlled characteristics (e.g., shape, pore size, roughness). Clinical and radiographical findings at any time point. Histological findings of the healing process at any time point. Reported complications at any time point. Patient-reported outcomes (e.g., pain, esthetic satisfaction). Main study’s conclusions.

Studies	Three-Dimensionally Printed Dental Implant	Three-Dimensionally Printed Manufacturing-Controlled Characteristics	Clinical and Radiographical Findings	Histological Findings of the Healing Process	Reported Complications	Patient-Reported Outcomes	Main Conclusions
Animal Study							
Balamurugan P., [83] 2021 J Ambient Intell Human Comput Animal study (cadaver goat) Sample size: MD	Material: Ti Three-dimensional printing method: SLM Bone defect: MD Position: posterior mandible	Shape Abutment Microstructure Mechanical properties	MD	MD	MD	NA	Digital manufacturing and back-engineering software make it possible to manufacture dental devices capable of withstanding high chewing forces and offer more flexibility than conventional dental implants.
Chang Tu C., [84] 2020 J Formos Med Assoc Animal study (white rabbits) Sample size: n.20	Material: Ti6Al4V (n.20) Three-dimensional printing method: laser sintering (n.20) Bone defect: MD Position: distal femoral condyle (n.20)	Shape Design Pore size Mechanical properties	MD	After 12 months Trabecular thickness and bone density were high. The trabecular thickness and porosity increased over time, reflecting the physiological bone remodeling process.	MD	NA	Three-dimensionally printed dental implants should be promising devices for GBR and the reconstruction of large bone defects due to failed implants.
Li L., [86] 2020 Materials (Basel) Animal study (beagle) Sample size: n.4	Material: Ti (n.4) Three-dimensional printing method: direct metal laser melting (n.4) Bone defect: MD Position: posterior mandible (n.4)	Shape Design Microstructure Pore size	After 3 months Implant stability: ranges from 71.18 ± 5.96 to 82.62 ± 4.09	After 3 months No evidence of inflammatory response, except for two implants. New bone was found in the threads and lattice structures of 3D printed implants.	Implant failure (n.2)	NA	Three-dimensionally printed dental implants with spikes had a comparable implant stability to 3D printed dental implants without spikes at 3 months. A 3D printed implant without spikes and conventional implants has comparable stability and bone-to-implant contact.
Tedesco J., [85] 2017 Int J Dent Animal pilot study (rabbit) Sample size: n.12	Material: Ti6Al4V (n.12) Three-dimensional printing method: direct melting laser sintering (n.12) Bone defect: MD Position: tibial metaphysis (n.12)	Shape Diameter Length Roughness	After 1 month: The newly regenerated bone accounted for 50% of the total length of the bone. After 3 months: The newly regenerated bone accounted for 55% of the total length of the bone.	After 1 month: The bone was actively remodeled around dental implants, and multinucleated osteoclast and hypertrophic osteoblast were found. No debris was detected. After 3 months: The bone was still actively remodeled around dental implants, but more mature bone morphology was detected. A layer of bone-covered dental implant surface was obtained.	MD	NA	A 3D printed dental implant provides a means for innovative designs with inherent implant surfaces, which enhance osseointegration in rabbits for up to twelve weeks. Dual-stemmed 3D printed dental implants showed successful bone–impact contact and bone growth similar to conventional or other 3D printed implant designs up to twelve weeks.
Wang H., [87] 2019 Mater Sci Eng C Mater Biol Appl Animal study (rabbits) Sample size: n.36	Materials: Ti and Tantalum (n.36) Three-dimensional printing method: SLM (n.36) Bone defect: MD Position: femur (n.36)	Shape Design Pore size Mechanical properties	MD	After 2 weeks Bone growth along the implant pores was found. After 4 weeks Bone growth was observed also in the center of implant pores.	MD	NA	Porous tantalum is a promising material for GBR.
In vitro Study							
Binobaid A., [89] 2024 J Mech Behav Biomed Mater In vitro study	Materials: lattice structure, zirconia, and calcium silicate Three-dimensional printing method: digital light processing	Shape Length (ultra-short) Material combination ratio Pore size Mechanical properties Biocompatibility	NA	NA	The lattice structure with a 300 µm pore size greatly influenced the implant’s mechanical properties and is not suitable for dental implants.		The mechanical properties of dental implants in zirconia/calcium silicate are similar to those of dental implants in Ti6Al4V and of cancellous bone.
Sonaye S.Y., [88] 2022 J Mech Behav Biomed Mater In vitro study	Material: PEEK Three-dimensional printing method: fused filament	Shape Implant–abutment connection Mechanical properties Aging properties Thermal properties	NA	NA	MD	NA	Three-dimensionally printed dental implants have the potential to be highly translational, with high resolution and mechanical properties even under axial forces. Personalized treatment with 3D printed dental implants requires low economic costs and time.

Abbreviations: three-dimensional, “3D”; number, “n”; titanium, “Ti”; aluminum, “Al”; vanadium, “V”; poly-ether-ether-ketone, “PEEK”; selective laser melting, “SLM”; missing data, “MD”, not applicable, “NA”.
